# Why do adults with dyslexia have poor global motion sensitivity?

**DOI:** 10.3389/fnhum.2013.00859

**Published:** 2013-12-12

**Authors:** Elizabeth G. Conlon, Gry Lilleskaret, Craig M. Wright, Anne Stuksrud

**Affiliations:** Griffith Health Institute, School of Applied Psychology, Griffith UniversityGold Coast, QLD, Australia

**Keywords:** coherent motion, dyslexia, temporal recruitment, perceptual contrast effect, attention

## Abstract

Two experiments aimed to determine why adults with dyslexia have higher global motion thresholds than typically reading controls. In Experiment 1, the dot density and number of animation frames presented in the dot stimulus were manipulated because of findings that use of a high dot density can normalize coherence thresholds in individuals with dyslexia. Dot densities were 14.15 and 3.54 dots/deg^2^. These were presented for five (84 ms) or eight (134 ms) frames. The dyslexia group had higher coherence thresholds in all conditions than controls. However, in the high dot density, long duration condition, both reader groups had the lowest thresholds indicating normal temporal recruitment. These results indicated that the dyslexia group could sample the additional signals dots over space and then integrate these with the same efficiency as controls. In Experiment 2, we determined whether briefly presenting a fully coherent prime moving in either the same or opposite direction of motion to a partially coherent test stimulus would systematically increase and decrease global motion thresholds in the reader groups. When the direction of motion in the prime and test was the same, global motion thresholds increased for both reader groups. The increase in coherence thresholds was significantly greater for the dyslexia group. When the motion of the prime and test were presented in opposite directions, coherence thresholds were reduced in both groups. No group threshold differences were found. We concluded that the global motion processing deficit found in adults with dyslexia can be explained by undersampling of the target motion signals. This might occur because of difficulties directing attention to the relevant motion signals in the random dot pattern, and not a specific difficulty integrating global motion signals. These effects are most likely to occur in the group with dyslexia when more complex computational processes are required to process global motion.

## INTRODUCTION

Dyslexia is a neurobiological disorder that can affect multiple brain areas (Stein, 2001; Shaywitz et al., 2003; Vidyasagar and Pammer, 2010). Adults with dyslexia show evidence of poor phonological awareness (Wilson and Lesaux, 2001), slower processing speed (Laasonen et al., 2001; Conlon et al., 2011; Stenneken et al., 2011), poor spelling (Bruck, 1990) and reduced comprehension (Conlon and Sanders, 2011). Although researchers agree on the characteristics that distinguish adults with good and poor reading skills, there is less agreement found concerning the visual processes that are impaired in this group.

Evidence that some individuals with dyslexia have a sensory processing deficit isolated to the magnocellular (M) and/or dorsal visual streams has been reported over many years (Lovegrove, 1993; Habib, 2000; Stein, 2001). Groups with dyslexia have poorer temporal contrast sensitivity than controls but do not have poorer spatial contrast sensitivity, measured in the parvocellular visual stream (Lovegrove et al., 1986). Difficulties replicating these findings (Amitay et al., 2002; Williams et al., 2003) and evidence that poorer sensitivity is found only with use of methodologies that require either sequential processing (Ben-Yehudah et al., 2001) or prior adaptation to a stimulus (Johnston et al., 2008), indicates that groups with dyslexia have a visual sensory processing deficit only when performing tasks that use complex computational processes. The evidence indicates that these processes are particularly impaired in the medial temporal area (MT) of the dorsal stream (Cornelissen et al., 1995; Talcott et al., 2000; Hansen et al., 2001; Conlon et al., 2004, 2009, 2012; Wilmer et al., 2004; Roach and Hogben, 2007; Benassi et al., 2010).

Evidence supporting the hypothesis of poorer performance of groups with dyslexia on computationally complex tasks is obtained from studies that have used methodologies that require discrimination of speed or the direction of global motion at MT. Reduced sensitivity is found in groups with dyslexia than in typically reading controls on these tasks (Cornelissen et al., 1995; Demb et al., 1998a; Raymond and Sorensen, 1998; Talcott et al., 2000; Hansen et al., 2001; Conlon et al., 2004, 2009; Wilmer et al., 2004; Wright and Conlon, 2009; Benassi et al., 2010). Convergent evidence of poorer performance in groups with dyslexia on speed, contrast and direction discrimination tasks has been obtained using electrophysiological (Schulte-Körne et al., 2004; Schulte-Körne and Bruder, 2010; Jednoróg et al., 2011) and functional MRI technology in which reduced neural activation has been found in these groups compared to controls (Eden et al., 1996; Demb et al., 1998b; Ben-Shachar et al., 2007).

Although there is compelling evidence for reduced efficiency when processing these complex stimuli in individuals with dyslexia, there have also been failures to replicate the effects found (Amitay et al., 2002; Hill and Raymond, 2002; Ramus et al., 2003; Reid et al., 2007). One explanation of these inconsistent findings concerns the extent that individuals with dyslexia can capture or sample the relevant motion signals for further processing. Using the global motion task, the aim of the experiments conducted was to determine whether coherent motion thresholds in groups with dyslexia would systematically change with presentation of stimuli that either increase or decrease the probability that coherent motion will be detected in the stimulus used.

The perception of global motion is commonly assessed using an apparent motion task generated with a random dot kinematogram (RDK) containing signal and noise dots (Newsome and Paré, 1988). This process occurs in the MT area of the dorsal stream (Thakral and Slotnick, 2011). The signal dots move in a single direction while noise dots move randomly. The RDK contains a series of single animation frames in which apparent motion is generated by presenting the dots in different locations in the RDK, and then presenting the series of stimuli rapidly and sequentially. Signal dots must be extracted from the noise dots and then integrated to form a global perception of motion (Raymond, 2000). The minimum percentage of signal dots needed for accurate perception of global motion is defined as the “motion coherence threshold.” The lower the proportion of signal dots needed to reach coherence threshold, the greater the sensitivity of the visual system to global motion.

Experimenters can systematically increase or decrease coherence thresholds by manipulating the stimulus parameters used to generate global motion. For example, increasing the number of animation frames presented on a single trial reduces coherence thresholds (McKee and Welch, 1985). This effect is known as temporal recruitment (Raymond and Isaak, 1998) and occurs because cell groups in the dorsal visual stream that are sensitive to direction of motion have increased opportunity to detect and integrate the signal dots across time with presentation of more animation frames. This allows for greater co-operation between stimulated motion analysers (Raymond and Isaak, 1998; Snowden and Braddick, 1989), which increases the probability that global motion will be detected.

Although there is evidence for temporal recruitment in good readers, the evidence for its influence on coherence thresholds in groups with dyslexia is limited. In one study, coherence thresholds were significantly lower in a group with dyslexia with presentation of ten animation frames (duration of 333 ms) when compared to four (duration of 133 ms; Hill and Raymond, 2002). However, in a second study, temporal recruitment had no influence on coherence thresholds in a group with dyslexia (Raymond and Sorensen, 1998). One important difference between the two studies was the dot density used to generate the RDK.

Dot density is the number of dots presented per degree of visual angle (dots/deg^2^). This parameter has little influence on coherence thresholds in typical readers (Barlow and Tripathy, 1997). However, increasing the dot density can reduce coherence thresholds in groups with dyslexia (Talcott et al., 2000). In a previous study that manipulated the dot density in a RDK, Talcott et al. (2000) found that coherence thresholds were higher in a group with dyslexia than in controls when the dot density was 9 dots/deg^2^ or less. No group differences in coherence thresholds were found when the dot density was 12.2 dots/deg^2^. Increasing the dot density increases the number of motion signals that can be sampled in a limited area in space. Previous research has concluded that directionally selective cells in the dorsal stream are fewer and more sparsely distributed in individuals with dyslexia (Galaburda and Livingstone, 1993; Stein and Walsh, 1997; Talcott et al., 2000). In addition, visual evoked potentials (VEP) are attenuated with presentation of coherent motion, but not by presentation of noise only (Schulte-Körne et al., 2004). On this basis increasing the dot density in a RDK might increase the probability that these sparsely distributed cell groups can capture and integrate motion signals (Talcott et al., 2000). This might explain the reported findings of no reader group differences on global motion tasks that have used high dot densities (Hill and Raymond, 2002; Edwards et al., 2004).

In most studies that have investigated global motion processing in dyslexia, a single task has been conducted to determine whether the groups with and without dyslexia differ on coherence thresholds. Investigation of the influence of the stimulus parameters used has been limited. Experiment 1 aims to systematically manipulate the number of animation frames presented and the dot density in the RDK, thereby determining the influence of these parameters on coherence thresholds in groups with dyslexia and controls.

Additional parameters found to systematically influence global motion thresholds in groups with dyslexia are the contrast or color of the signal and noise dots presented in the RDK, and the use of a pre-cue as an attention aid. When the signal and noise dots are the same color and contrast, groups with dyslexia have higher coherence thresholds than controls (Cornelissen et al., 1995; Talcott et al., 1998, 2000). When the signal dots are of either a higher contrast or presented in a different color to the noise dots, no group differences in coherence thresholds are found (Sperling et al., 2006; Conlon et al., 2012). Using this methodology, the influence of the noise dots is reduced, therefore allowing more efficient sampling of the signal dots in the global stimulus. One explanation of this effect is that presentation of stimuli of higher contrast produce increased excitation in the cell groups stimulated, allowing automatic exclusion of the noise dots in the RDK (Edwards et al., 1996; Croner and Albright, 1997; Martinez-Trujillo and Treue, 2002). When the stimuli of higher contrast are the target motion signals, there is a greater probability that these stimuli will be sampled by the visual system, particularly when competing with lower contrast, lower energy stimuli for sampling.

Relative to conditions in which the signal and noise dots in the RDK are of equal contrast, individuals with dyslexia have higher coherence thresholds than controls when the signal dots are presented at a lower contrast than the noise dots (Conlon et al., 2012). This occurs because the high contrast, high energy noise dots mask the lower contrast, lower energy signal dots, reducing the capacity of the group with dyslexia to sample the available signal dots. However, if the signal dots are of a lower contrast than the noise dots and a pre-cue is presented alerting participants that the low contrast dots contain the coherent motion signals, no reader group differences in coherence thresholds are found. This occurred because the pre-cue resulted in lower coherence thresholds for the group with dyslexia but had no influence on the control group (Conlon et al., 2012). These findings are consistent with studies that have reported that the use of a pre-cue can also increase accuracy in visual search tasks that contain multiple stimuli in groups with dyslexia (Hawelka and Wimmer, 2008; Moores et al., 2011). One explanation of these findings is obtained from physiological data. Sensitivity to motion at MT in single cell recordings can be influenced by the attentional state of the receptive field (Treue and Maunsell, 1996; Treue and Trujillo, 1999). Using functional MRI technology, these findings have been extended to demonstrate an increased level of activation at MT in the human visual system on the basis of manipulations of attention to specific stimulus attributes (O’Craven et al., 1997; Buchel et al., 1998), for example, the speed changes in a motion stimulus. Behaviourally, directing attention in this way increases the length of the motion aftereffect, relative to passive viewing conditions (Buchel et al., 1998). The increased activity at MT occurs because of a top-down feedback loop from the posterior parietal cortex (PPC). This feedback loop acts to modulate sensory performance. In groups with dyslexia there is now substantial evidence of impairment in attention processing at the level (Vidyasagar, 1999; Lallier et al., 2010; Vidyasagar and Pammer, 2010).

A problem with the studies that have manipulated contrast or color or used a pre-cue is that the signal dots differed from noise in terms of motion, color and contrast. It may therefore have been the influence of contrast or color, rather than the availability of the target signal dots that led to systematic increases and decreases in coherence thresholds in groups with dyslexia.

The impact of stimulus parameters that can systematically influence coherence thresholds, while allowing the signal and noise dots to differ only on the direction of motion requires further investigation. One task that has been found to produce systematic changes in coherence thresholds by using motion alone is the perceptual contrast effect (Raymond and Isaak, 1998). Relative to a baseline condition in which coherence thresholds are obtained using a static prime, coherence thresholds are increased (i.e., sensitivity is reduced) in typical readers when a briefly presented, fully coherent motion prime is presented before a partially coherent test stimulus. This effect occurs provided the prime and test stimulus have the same direction of motion. When motion detectors responsive to the same direction of motion are stimulated by both the prime and test stimuli, the highly salient fully coherent prime reduces the visibility of the signal dots presented in the test stimulus because of the dramatic change in coherence of the prime and test. In fact, if presentation of the final two frames in a sequence of fully coherent motion is presented as partially coherent, these frames are not detected by participants (Raymond and Isaak, 1998). In contrast, coherence thresholds are reduced relative to the baseline condition when the prime and test have opposite directions of motion (Raymond and Isaak, 1998). The latter result might have occurred because the visibility of noise dots that matched the direction of motion in the prime was reduced, so decreasing the proportion of effective noise dots available for processing in the test RDK.

In explanation of their results, Raymond and Isaak (1998) argued that the prime was viewed as an object, so could disrupt processing of subsequently presented stimuli by reducing the efficiency of visual selection processes, if the prime and test had the same motion characteristics. These results cannot be explained by adaptation because the duration of the prime was less than 100 ms and the interstimulus interval (ISI) between the prime and test stimuli had no influence on thresholds (Raymond and Isaak, 1998; Glasser et al., 2011). Importantly, the only difference between the stimuli presented were the motion attributes of the prime. Experiment 2 will use the perceptual contrast effect to determine whether coherence thresholds can be systematically increased or decreased in individuals with dyslexia.

The overall aim of the experiments conducted in this study was to determine if using different methodologies can vary the strength of the motion signals used in a RDK, thus leading to subsequent systematic changes in coherence thresholds in groups with and without dyslexia. In the first experiment, the dot density and number of animation frames presented in a RDK were manipulated. In Experiment 2, coherence thresholds were obtained after brief exposure to a fully coherent prime moving in either the same or opposite direction of motion to the partially coherent test stimulus.

### EXPERIMENT 1

The effect of dot density and temporal recruitment on coherence thresholds in groups of adults with or without dyslexia was investigated. Two dot densities, high (14.15 dots/deg^2^) and low (3.54 dots/deg^2^) were used. These dot densities were selected based on findings that no reader group differences in coherence thresholds are found at dot densities of 12.2 dot/deg^2^ or greater (Talcott et al., 2000; Hill and Raymond, 2002). Temporal recruitment was manipulated by presenting each dot density condition for five (total duration, 83 ms) or eight (total duration, 133 ms) animation frames. These parameters were selected because Hill and Raymond (2002) found no reader group difference on a global motion processing task when dot density was high (45 dots/deg^2^) and four animation frames (total duration, 133 ms) were presented.

If increasing dot density alone provides a sufficient increase in the capacity of the group with dyslexia when sampling the signal dots, no group difference in coherence thresholds were expected with presentation of a RDK with high dot density. This was expected to occur regardless of the number of animation frames presented. The group with dyslexia were expected to have higher coherence thresholds than the control group when dot density was low. If temporal recruitment effects are only found in the group with dyslexia in the high dot density condition, lower coherence thresholds were expected with presentation of the high dot density condition in which eight animation frames were presented. In the low dot density condition, no influence of the number of animation frames presented was expected.

## MATERIALS AND METHODS

### PARTICIPANTS

There were 21 individuals with dyslexia (*M*_age_ = 23.64 years; *SD* = 6.4) and 22 typically reading controls (*M*_age_ = 18.64 years; *SD* = 3.33). All participants had English as a first language and normal or corrected to normal visual acuity. Due to associations found in previous studies between visual discomfort and global motion processing, participants with a high score on the Visual Discomfort Scale were excluded (Conlon et al., 1999, 2009). Individuals with dyslexia were recruited from the University disability office, the laboratory register and from advertising. Typical readers were obtained from the student participant pool. All procedures were conducted in accordance with the University human research ethics committee that approved this project.

The criteria used to define adults with dyslexia were based on those used previously (Conlon et al., 2004, 2009, 2011, 2012; Conlon and Sanders, 2011). Individuals with dyslexia reported a history of reading difficulties and had standard word reading scores below average on the Wide Range Achievement Test – 3rd Edition (WRAT-3; Wilkinson, 1993). This test consists of 42 words of increasing difficulty and has internal consistencies of 0.90–0.95 for the age groups used in this study. A further criterion was that scores on the test of word reading efficiency (TOWRE; Torgesen et al., 1999) were below a standard score of 90.

Individuals with dyslexia also had scores at least two standard deviations (SD) below the mean of the control group on non-word and exception word reading tests. The non-word and exception word tests each had 25 items matched for word length. The internal consistencies for the non-word and exception word tests are .77 and .84 respectively. At least average ability as measured by the Block Design subtest from the WAIS-3 (Wechsler, 1998) was the final criterion.

Criteria for inclusion in the control group were word reading scores on the WRAT-3 and reading fluency scores on the TOWRE of at least a standard score of 105. Nonword and exception word reading test scores were at least 75%. At least average ability as measured by the Block Design subtest from the WAIS-3 (Wechsler, 1998) was the final criterion.

The group with dyslexia was significantly poorer than the control group on word reading, *t*(41) = 13.24, *p* < 0.001, non-word reading, *t*(41) = 10.61,* p *< 0.001, exception word reading, *t*(41) = 11.23, *p* < 0.001, and word reading fluency, *t*(41) = 12.24, *p* < 0.001 tests. No significant difference was found between groups on the measure of non-verbal ability used, *t*(41) = 0.238, *p* = 0.81. Both groups performed in the average range (see **Table 1**).

**Table 1:** Performance of the control (*n* = 22) and dyslexia (*n* = 21) groups on the reading and ability measures. Experiment 1.

	Control	Dyslexia
	Mean (SD)	Mean (SD)
WRAT reading (standard)	110.82 (4.35)	91.67 (5.12)
Non-words/25	22.32 (1.8)	14.43 (2.9)
Exception words/25	19.5 (1.8)	10.57 (3.2)
TOWRE total (standard score)	112.4 (6.8)	83.48 (8.6)
Non-verbal ability (scaled)	12.54(3.0)	12.52(2.8)

### STIMULI

Stimuli for the global motion task were generated using the Cambridge Research Systems hardware and Operating System Software, VSG Version 2/5. Stimuli were displayed on a 21 inch Hitachi HM-4721-D monitor with a resolution of 800 × 600 pixels, and a vertical screen refresh rate of 120 HZ.

The RDK contained either 100 (low dot density: 3.54 dots/deg^2^) or 400 (high dot density: 14.15 dots/deg^2^) white dots (luminance: 20 cd/m^2^) presented on a black background (luminance: 0.54 cd/m^2^) displayed within a borderless area subtending 6° × 6° presented in the middle of the computer screen. The display size was chosen to avoid pursuit eye movements (Hill and Raymond, 2002). The velocity of the stimuli was 10.5°/s and the dots had a diameter of one pixel (0.35 mm). The duration of a single animation was 16.67 ms, with a dot lifetime of two animation frames (33.34 ms), after which the signal dots disappeared before being regenerated at a randomly selected stimulus location within the panel. A standard wrap around technique was used for the signal dots as they reached the side of the screen. The noise dots randomly changed position in a Brownian fashion. Stimuli were presented for either five (84 ms) or eight animation frames (133 ms).

For each of the experimental conditions there were two blocks of trials. Separate coherence thresholds were obtained for each block. The adaptive psychophysical procedure used to estimate coherence thresholds was a three-down, one-up staircase with eight reversals. After three correct responses, coherence was halved, and after each incorrect response coherence was doubled. This allowed for an estimation of the coherence value needed to obtain a correct response on 79% of the trials (Kaernbach, 1991). Participants selected the direction of motion, left or right at the completion of each trial. Geometric mean thresholds were combined across both blocks of trials to obtain an overall estimate of coherence thresholds. The starting coherency was 50% in each condition.

Response bias was determined by presenting trials at 1% coherence at least once every five trials. Participants were expected to respond randomly to these trials, with about half the responses being to the left and half to the right. Response bias was evaluated by obtaining a percentage score for the proportion of left responses to these trials. No significant group differences in response bias were found (dyslexia, *M* = 46%, *SD* = 12.36; control, *M* = 44%, *SD* = 11.07), *t*(42) = 0.676, *p* = 0.503.

### PROCEDURE

Each participant was assessed for reading ability. This assessment was followed by a separate session in a darkened laboratory in which coherence thresholds were obtained. Viewing was binocular with natural pupils and the viewing distance of 57 cm was controlled with a chin rest. A block of 20 practice trials was presented prior to each of the four conditions. Each trial began with presentation of a fixation cross which was replaced after 150 ms with the RDK. Participants responded to the direction of motion by depressing either the left or right button on the Cambridge Research Systems CB-2 response box at the end of each trial. A new trial was automatically triggered after each response. The order of presentation of the experimental conditions was counterbalanced between and within groups.

## RESULTS

**FIGURE 1:**
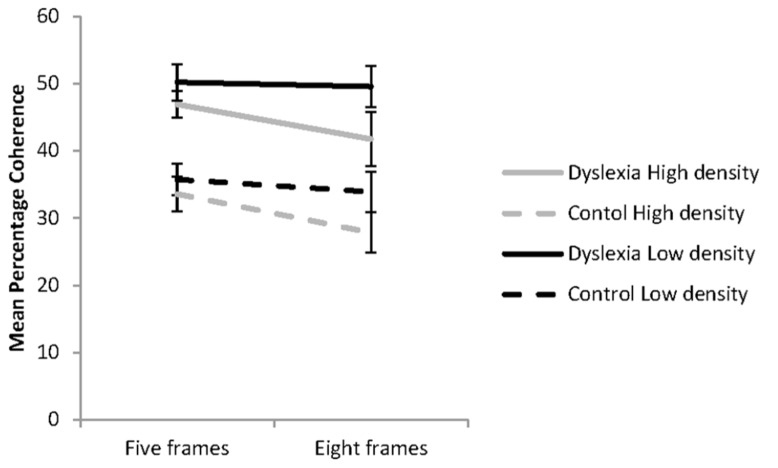
**Coherent motion thresholds for the effects of dot density and the number of animation frames presented for the groups with (*n* = 21) and without dyslexia (*n* = 22) in each of the four experimental conditions**. Error bars represent ±1 standard error.

The influence of dot density and the number of animation frames presented on coherence thresholds for the reader groups is shown in **Figure 1**. These data were analyzed using a 2 (group: dyslexia or control) × 2 (dot density: low or high) × 2 (animation frames: five or eight) mixed factorial ANOVA. The assumptions of the analysis were met. A significant main effects was found for reader group, *F*(1, 41) = 15.80, *p* < 0.001; $\eta_{p}^{2}$ = 28. Regardless of the condition, the group with dyslexia (*M* = 47.09; *95%* *CI* = 41.87–52.03) had higher coherence thresholds than controls (*M* = 32.74; *95%* *CI* = 27.65–37.84). Significant main effects were also found for dot density, *F*(1, 41) = 18.81, *p* < 0.001; $\eta_{p}^{2}$ = 0.31, and the number of animation frames presented, *F*(1, 41) = 10.66, *p* = 0.002; $\eta_{p}^{2}$ = 0.21. These were modified by a significant interaction between dot density and the number of animation frames presented, *F*(1, 41) = 8.68, *p* = 0.005, $\eta_{p}^{2}$ = 0.17. There were no other significant interactions found.

The influence of dot density and the number of animation frames presented on coherence thresholds was investigated with simple effects analysis. In the five-animation frame condition, dot density, high or low had no influence on coherence thresholds, *F*(1, 41) = 3.97, *p* = 0.069, $\eta_{p}^{2}$ = 0.08. Significantly lower coherence thresholds were found with presentation of the high compared to the low dot density stimuli for the eight animation frame condition, *F*(1, 41) = 32.29, *p* < 0.001, $\eta_{p}^{2}$ = 0.44. When dot density was low there was no evidence of temporal recruitment found, *F*(1, 41) = 0.73, *p* = 0.396, $\eta_{p}^{2}$ = 0.02. However, when dot density was high, coherence thresholds were significantly lower with presentation of eight than five animation frames, *F*(1, 41) = 28.8, *p* <0.001, $\eta_{p}^{2}$ = 0.41, showing the influence of temporal recruitment. The percentage reduction in coherence thresholds in the high dot density condition with presentation of the higher number of animation frames was 7.5% for the group with dyslexia and 6.1% for the control group, indicating similar effects of temporal recruitment in each reader group.

## DISCUSSION

It was expected that manipulating the dot density and the number of animation frames presented in a single trial of the global motion task would systematically increase or decrease the capacity of the reader groups to efficiently sample the motion signals present in the RDK. The findings from the study were partially consistent with our hypotheses. Regardless of reader group, lower coherence thresholds were found with presentation of the high dot density condition when presented for eight animation frames, showing temporal recruitment effects. In the low dot density condition, temporal recruitment effects were not found. Across all conditions presented, the group with dyslexia had higher coherence thresholds than the control group. These findings are partially consistent with our hypotheses for the group with dyslexia only.

Previous studies have found that presentation of a RDK with a high dot density promotes increased processing efficiency in the group with dyslexia (Hill and Raymond, 2002; Edwards et al., 2004) because of the increased capacity to sample the signal dots presented within a limited area in space (Talcott et al., 2000). The findings of the current experiment indicate that the combination of high dot density and eight-animation frames used was not sufficient to normalize coherence thresholds in the group with dyslexia. The previous study that found no reader group differences in coherence thresholds when four animation frames (133 ms) were presented used a higher dot density (45 dots/deg^2^; Hill and Raymond, 2002) than that used in the current study. In addition, when using a dot density of 12.2 dot/deg^2^ one previous study found no reader group differences on the global motion task when stimulus durations of 900 ms were used (Talcott et al., 2000). These results indicate that dot density alone, unless very high, cannot normalize coherence thresholds in the group with dyslexia.

Lower coherence thresholds were found in the group with dyslexia in the high dot density condition and when the RDK was presented for eight animation frames. These results indicate that given sufficient signal dots captured when the higher dot density was used, normal temporal recruitment is found in the group with dyslexia. This result is consistent with the findings of Hill and Raymond (2002). The group with dyslexia can integrate motion signals over time, given sufficient motion samples from the high dot density condition. Findings that temporal recruitment did not occur in the low dot density condition support this conclusion, a result consistent with a previous study (Raymond and Sorensen, 1998).

Together these findings indicate that integration of the signal dots over time in the global motion task relies on the observer’s capacity to extract sufficient signal dots from noise. A minimum level of energy in the motion signals may be required. This could be obtained with presentation of a high dot density, a greater number of animation frames or a combination of both. These findings might indicate that with sufficient signal energy to stimulate adequate neural activity in the less efficient dorsal stream of the group with dyslexia, the computation of the direction of global motion becomes more efficient.

There was no evidence of temporal recruitment in the low dot density condition for either reader group. Although these results were expected for the group with dyslexia, temporal recruitment effects were expected in the control group in this condition (Raymond and Sorensen, 1998; Talcott et al., 2000). These results indicate that presentation of the five frame stimulus in which the total stimulus duration was 84ms increased the perceptual difficulty of the stimulus beyond a level that even a well-functioning system could utilize when the dot density was low (Braddick, 1995). In addition, the duration of a single animation frame was short. Although the dot life-time of 33 ms was consistent with that used in previous studies (Raymond and Sorensen, 1998; Hill and Raymond, 2002), the frame duration was below 20 ms. In this case, a greater number of animation frames might have been needed to reach asymptotic motion thresholds (Snowden and Braddick, 1989).

The critical findings obtained from Experiment 1 are that coherent motion thresholds can be reduced in groups with and without dyslexia by increasing both dot density and the number of animation frames in a RDK. However, none of the experimental manipulations led to coherent motion thresholds being normalized in the group with dyslexia who had higher thresholds than controls in all conditions.

### EXPERIMENT 2

Coherent motion thresholds in groups with dyslexia can be systematically increased by presenting signal dots at a lower contrast than the noise dots, and decreased by presenting signal dots at a higher contrast or different color to the noise dots (Sperling et al., 2006; Conlon et al., 2012). These effects are found because the higher contrast signals are the most salient so are preferentially processed in the human visual system (Edwards et al., 1996; Croner and Albright, 1997). In Experiment 2, manipulating motion only, we aimed to determine if coherence thresholds could be systematically increased and decreased in a group with dyslexia using a fully coherent prime presented in either the same or opposite direction of motion to the test stimulus.

It was expected that presentation of a fully coherent prime before the test stimulus would reduce the visibility of the signal dots in the partially coherence test stimulus in a similar way to that found when noise dots are presented at a higher contrast to the signal dots in the RDK. This would occur because of the reduced salience of the coherent motion in the partially coherent test, relative to the highly salient fully coherent prime. We expected that the threshold elevation found in a group with dyslexia would be greater than that found in the control group. However, if presenting a fully coherent prime in the opposite direction to the partially coherent test stimulus facilitates global motion processing in a group with dyslexia in a similar way to that found when presenting signal dots at a higher contrast to the noise dots, it was expected that no group difference in coherence thresholds would be found. In the baseline condition in which a stationary test stimulus was presented, higher coherence thresholds were expected in the group with dyslexia than for controls.

## MATERIALS AND METHODS

### PARTICIPANTS

There were 20 participants, 10 with dyslexia (*M*_age_ = 22.11 years, *SD* = 3.49) and 10 normally reading controls (*M*_age_ = 21.9 years; *SD* = 4.12). They were obtained using the same procedures as Experiment 1. No participant took part in both studies. All participants had a history of dyslexia, English as a first language and normal or corrected to normal visual acuity. The study had approval from the Human Research Ethics Committee. Group classification procedures were the same as Experiment 1 (see **Table 2**).

**Table 2:** Performance of the control (*n* = 10) and dyslexia (*n* = 10) groups on the reading measures. Experiment 2.

	Control	Dyslexia
	Mean (SD)	Mean (SD)	*t*-test
WRAT reading (standard)	115.6 (4.35)	94.1 (5.4)	*t*(18) = 10.88, *p* < 0.001
Non-words/25	24.0 (0.89)	15.5 (3.4)	*t*(18) = 6.85, *p* < 0.001
Exception words/25	21.9 (0.78)	11.4 (2.9)	*t*(18) = 10.36, *p* < 0.001
Non-verbal ability (scaled)	12.1 (0.87)	11.4(1.90)	*t*(18) = 1.06, *p* = 0.303

### STIMULI AND APPARATUS

**FIGURE 2:**
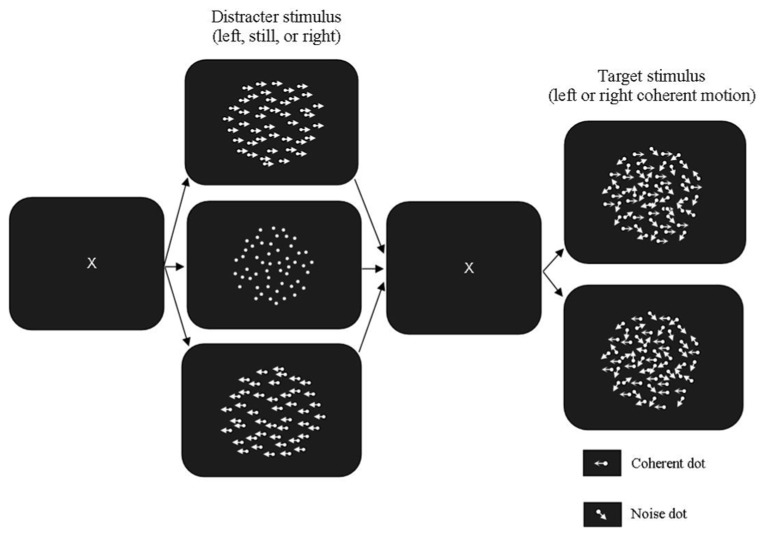
**Motion Segmentation Task used in Experiment 2**. The fully coherent prime was presented in either the same or opposite direction to the partially coherent test. The stationary prime was used as a control condition. The blank stimulus between the prime and test was presented for 96 ms and the test stimulus was presented for 160 ms (10 animation frames).

The apparatus and adaptive psychophysical procedure used were the same as those used in Experiment 1. In the global motion task, the RDK used as both the prime and the test had 300 white dots (luminance: 20 cd/m^2^) presented on a dark background (luminance: 0.54 cd/m^2^). The stimulus was displayed within a borderless area subtending 13.35° × 13.35° presented in the middle of the computer screen. Dot density was 3.83 dots/deg^2^ and dot life time was three animation frames (50 ms). The dot life time was increased from Experiment 1 from the results of pilot testing. The motion primes were presented at 100% coherence, with one moving to the left and the other moving to the right. The baseline control stimulus was a stationary RDK with no dot displacement. Each of these stimuli was presented for 96 ms. When extinguished these were replaced with a blank low luminance field (0.54 cd/m^2^) for 32 ms. This field was replaced with the partially coherent test stimulus, which was presented for 10 animation frames (160 ms). The method used is shown in **Figure 2**.

In each condition, the task was to determine whether the direction of coherent motion was to the left or to the right. Beginning coherence in all conditions was 25%. Two blocks of trials were presented for each condition. Threshold estimates were based on six threshold reversals for each block of trials. The geometric mean coherence thresholds were obtained for each block of trials. These were averaged to determine coherence thresholds for each condition.

### PROCEDURE

The global motion task was conducted in a separate session after assessment of reading ability. Testing took place in a darkened laboratory. Viewing distance of 57 cm was controlled with a chin rest. Viewing was binocular with natural pupils. Participants were instructed to judge whether the dots presented on the screen were moving to the left or the right. A block of 20 practice trials was followed by the experimental trials. Participants registered the direction of coherent motion by depressing the left or right keys on the response box at the end of each trial. A new trial began automatically after a response.

## RESULTS

**FIGURE 3:**
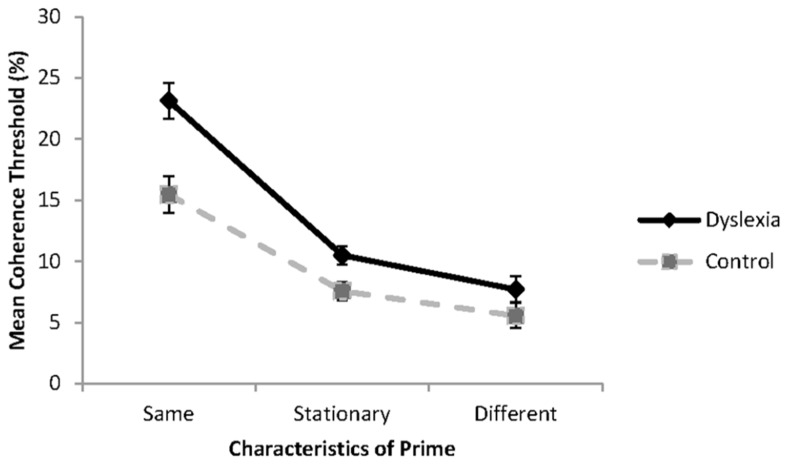
**Global motion coherence thresholds for group with dyslexia (*n* = 10) and controls (*n* = 10) when the probe and prime are presented in the same direction (same), in opposite directions (different) or when the prime is a stationary control stimulus**. Standard error bars represent ±1 standard error.

The results of the experiment are shown in **Figure 3**. The impact of the prime on threshold performance was analyzed using a 3 (condition: same, different, baseline) × 2 (group: dyslexia or control) mixed factorial ANOVA. All the assumptions of the analysis were met. Significant main effects were found for condition, *F*(2, 36) = 113.8, *p* < 0.001, $\eta_{p}^{2}$ = 0.86, and reader group, *F*(1, 18) = 11.80, *p* = 0.003, $\eta_{p}^{2}$ = 0.40. These effects were modified with a significant reader group by condition interaction, *F*(2, 36) = 5.61, *p* = 0.008, $\eta_{p}^{2}$ = 0.24.

Inspection of **Figure 3** shows that as expected, highest coherence thresholds were found when the prime and test were presented in the same direction of motion, and lowest coherence thresholds were found when the prime and test were presented in opposite directions of motion. Simple effects analysis revealed that the group with dyslexia had significantly higher coherence thresholds than the control group when the prime and test were presented in the same direction of motion, *F*(1, 18) = 13.34, *p* = 0.002, $\eta_{p}^{2}$ = 0.43, and in the baseline condition, when the prime was a stationary stimulus, *F*(1, 18) = 7.69, *p* = 0.013, $\eta_{p}^{2}$ = 0.30. No significant differences were found between reader groups when the prime and test were presented in opposite directions of motion, *F*(1, 18) = 2.07, *p* = 0.167, $\eta_{p}^{2}$ = 0.10. These results are consistent with the hypotheses of the study.

To determine to what extent the prime influenced coherence thresholds in each of the groups relative to the baseline condition, difference scores were obtained for each of the primed thresholds. Coherence thresholds were elevated by 12.63% (*SD* = 3.81%) for the group with dyslexia and 7.87% (*SD* = 3.60%) for the control group when the motion of the prime and the test was presented in the same direction. The increase in threshold was significantly greater for the group with dyslexia than for the control group, *t*(18) = 2.86, *p* = 0.010. When the prime and test were presented in opposite directions of motion, thresholds were enhanced by 2.76% (*SD* = 1.74%) for the group with dyslexia, and by 2.03% (*SD* = 1.48%) for the control group. The degree to which coherence thresholds were reduced, did not differ between the reader groups, *t*(18) = 0.68, *p* = 0.513, failing to support the hypothesis of greater facilitation in processing for the group with dyslexia. Although this change was sufficient for findings of a statistically significant threshold enhancement when compared to the baseline condition for the group with dyslexia, *t*(18) = 4.78, *p* < 0.001, the change was not sufficient to reveal reader group differences.

## DISCUSSION

It was expected that presentation of a prime in the same or opposite direction of motion to the test would systematically increase or decrease the capacity of the group with dyslexia when sampling the signal dots in the partially coherent test stimulus. The results of the study are consistent with our hypotheses. Relative to the baseline condition, for both reader groups, higher coherence thresholds were found when the prime and test were presented with the same direction of motion and lower coherence thresholds were found when the prime and test were presented with opposite directions of motion. These results replicate the original findings of Raymond and Isaak (1998).

The significantly greater increase in coherence thresholds found in both reader groups when the prime and test were presented in the same direction of motion is consistent with our hypothesis that exposure to the highly salient fully coherent prime would reduce the visibility of the coherent motion signals in the test stimulus. This effect could have occurred because the visibility of the coherent motion signals was reduced, temporarily reducing their salience relative to the noise dots. This effect would have reduced the capacity of each reader group when excluding the noise dots in the test RDK. In this condition, the group with dyslexia had greater difficulty than the control group sampling the signal dots presented in the partially coherent test, producing a greater processing disadvantage. These findings are similar to those obtained in a previous study in which the signal dots were presented at a lower contrast than the noise dots in a RDK (Conlon et al., 2012). One explanation of these results is the capacity of the group with dyslexia to sample the available motion signals was reduced more than that found in the control group because of undersampling of the signal dots presented.

No significant reader group differences in coherence thresholds were found when the prime and test were presented in opposite directions of motion. These results are similar to those obtained when the signal dots presented in the RDK were a higher contrast or different color to the noise dots (Sperling et al., 2006; Conlon et al., 2012). This result occurred because of the higher energy in the target signal dots (Croner and Albright, 1997; Martinez-Trujillo and Treue, 2002) allowing greater sampling of these stimuli over the lower contrast noise dots. When the prime and test were presented in opposite directions of motion, similar facilitation of coherence thresholds was found. When the direction of motion in the test was in the opposite direction to the prime, the salience of a proportion of the noise dots would have been reduced, which might have increased the probability that signal dots would have been sampled. Evidence supporting this conclusion is obtained from the influence of presentation of a prime containing noise dots only. Higher coherence thresholds were found in the test because some of the directions of motion in the prime also masked the motion signals in the test stimulus (Raymond and Isaak, 1998).

The last important finding from the experiment was that the group with dyslexia had higher coherence thresholds than controls in the baseline control condition when the prime was a stationary stimulus. This result is consistent with many studies that have shown evidence of a global motion processing deficit in groups with dyslexia (Cornelissen et al., 1995; Raymond and Sorensen, 1998; Talcott et al., 2000; Conlon et al., 2004). Overall the findings from the experiment indicate that presentation of a fully coherent prime can influence the size of coherence thresholds, by increasing or decreasing the proportion of signal dots that can be easily sampled by individuals with dyslexia. While the perceptual processing explanation presented here can explain the systematic changes in coherence thresholds found, and provides a sensory explanation of the effects for the group with dyslexia, attention mechanisms might also be implicated. These will be discussed in the following section.

## GENERAL DISCUSSION

The results of these experiments indicate that motion coherence thresholds in groups with dyslexia can be systematically increased or decreased when the capacity of these individuals to sample the signal dots in the RDK is manipulated. Increasing the dot density and number of animation frames used or presenting the prime and test stimulus in opposite directions of motion results in lower coherence thresholds for each reader group. Conversely, reducing the dot density and the number of animation used or presenting the prime and test in the same direction of motion produces higher coherence thresholds for each reader group. The efficiency of the computational processes needed when undertaking a global motion task in individuals with dyslexia will be discussed. The viability of explanations which include sensory processes only and those that include attention mechanisms will each be addressed.

When processing global motion two important computational processes are required, that of extracting signal from noise and integration of the extracted motion signals over space and time (Raymond, 2000). The results of the current study demonstrate that the efficiency with which individuals with dyslexia can sample the signal dots in the RDK and therefore efficiently perform these processes depends on the stimulus parameters and methodology used. The latent variable manipulated in both experiments was the strength or energy of the motion signals presented in the RDK. In Experiment 1, higher signal strength was produced by increasing the dot density and the number of animation frames over which the RDK was presented. In Experiment 2 signal strength in the test stimulus was manipulated by the direction of motion in the prime. In both experiments, lower coherence thresholds were obtained in the reader groups when stimuli producing the strongest motion signals were presented. Highest coherence thresholds were found in both experiments, when the strength of the signal dots in the RDK was weakest. In Experiment 1, this was presentation of stimuli with a low dot density, or presentation of stimuli presented for five animation frames and in Experiment 2, this was the condition in which the prime and test stimuli were presented in the same direction of motion.

Although the group with dyslexia had higher coherence thresholds than typical readers in all conditions of Experiment 1, evidence of normal temporal recruitment was found in the high dot density condition. Coherence thresholds for both reader groups were reduced by over 6% with presentation of the eight compared to the five frame condition. These results indicate normal temporal recruitment in the group with dyslexia when these individuals are able to sample sufficient motion signals. In a previous study that used a higher dot density than that used in the current study, normal temporal recruitment was found in the group with dyslexia. In addition, no significant reader group differences in coherence thresholds were found (Hill and Raymond, 2002). Together these results indicate two things. First, if dot density is sufficiently high, normal temporal recruitment occurs in the group with dyslexia because these individuals are able to sample sufficient motion signals to perform the integration process. Second, either a very high dot density or a combination of a high dot density and longer stimulus durations is needed to enable normal global motion processing in the group with dyslexia. In low dot density conditions, problems with temporal recruitment were found. This might occur because of the poorer capacity of individuals with dyslexia when sampling more motion signals presented more sparsely in space (Talcott et al., 2000; Stein, 2001, 2003). Electrophysiological studies in which lower activation was found with exposure to coherent motion but not to noise dots alone provides support for this explanation (Schulte-Körne et al., 2004; Jednoróg et al., 2011), indicating that the strength of the motion signals alone might be insufficient to promote efficient integration in the group with dyslexia.

Evidence that increased signal strength can normalize coherent motion processing in the group with dyslexia was also obtained in Experiment 2, where there were no significant group differences found when the prime and test stimuli were presented in opposite directions of motion. In this condition, the reduced salience of a proportion of the noise dots increased the efficiency of the signal extraction process, which in turn resulted in efficient integration of the extracted signals. These results are consistent with previous studies that have presented stimuli in which the noise dots were automatically excluded (Sperling et al., 2006; Conlon et al., 2012). It would be tempting to conclude that purely sensory processes are sufficient to potentially normalize coherent motion thresholds in groups with dyslexia, by facilitating noise exclusion. However, the impact of attention must also be considered.

Evidence that presentation of a fully coherent prime activates spatial attention has been found in an experiment similar to that conducted in Experiment 2, in which a transparent motion stimulus was used as the prime (Raymond et al., 1998). In a transparent motion task two fully coherent sheets of dots appear to move independently, each with orthogonal directions, for example motion moving leftward and upward. Prior to presentation of the prime a pre-cue alerted participants to the direction of motion in the transparent prime (horizontal or vertical) for which a judgment of motion direction was made. Presentation of the prime was followed by the partially coherent test stimulus, for which coherence thresholds were obtained. The results of the study found that for typical readers, if the attended direction of motion in the prime matched the direction of motion in the test, higher coherence thresholds were obtained. If the non-attended direction of motion of the prime matched the direction of motion of the test, lower coherence thresholds were found. These results were obtained only when the prime contained fully coherent motion, and not when arrows indicating the directions of motion were presented. These results indicate that selective attention is activated by the motion prime which influences coherence thresholds in the test, dependent on the allocation of attention to the prime (Raymond et al., 1998). These results are consistent with physiological data that has shown attention modulates activity in MT (O’Craven et al., 1997; Buchel et al., 1998). These results suggest that presentation of a single direction fully coherent prime also activated spatial attention when using the current methodology. Spatial attention may have modulated the response to the partially coherent test stimulus, dependent on the direction of motion in the prime. The time course of this activity might have been different for the group with dyslexia and the control group because of impaired attentional mechanisms in the group with dyslexia.

There is a growing body of research that has found groups with dyslexia have difficulties directing attention to rapidly presented stimuli (Vidyasagar and Pammer, 2010), shifting attention between stimulus sequences that are rapidly and sequentially presented (Hari and Renvall, 2001; Visser et al., 2004; Lallier et al., 2010) or orientating spatial attention (Facoetti et al., 2010). In the motion segmentation task (Experiment 2), two distinct stimulus events occurred rapidly and sequentially. First, the fully coherent prime was separated from the partially coherent test by an ISI of 32 ms. Spatial attention would have been automatically captured by the fully coherent prime, stimulating cell groups responsive to that direction of motion at MT (Martinez-Trujillo and Treue, 2002). With the rapid presentation of the test stimulus, attention had to be rapidly disengaged from the prime and directed at the test stimulus. If the group with dyslexia have difficulty rapidly disengaging attention from the prime and re-engaging attention on the test as suggested by Hari and Renvall (2001), the influence of the prime might be greater for the group with dyslexia than for the control group. When the prime and test were presented in the same direction of motion, difficulties disengaging attention from the prime might have added to the poor salience of the target motion signals in the test. This would have produced the much higher coherence thresholds found in this condition for the group with dyslexia than those found for the control group. Conversely, difficulties disengaging attention from the opposite direction prime might have increased the salience of the target motion signals in the test more for the group with dyslexia than for the control group, resulting in no significant group differences in coherence thresholds. This explanation raises the possibility that difficulties shifting attention are most apparent when stimuli to be processed stimulate cells groups in the same cortical area, so are task relevant. As no condition was presented using a blank prime, it is unknown if presentation of the stationary baseline condition also influenced thresholds. However, if difficulties shifting attention between the prime and test stimuli, partially account for the results obtained, presentation of longer ISIs between the prime and test, should reduce the processing disadvantage found when the prime and test are presented in the same direction. When the prime and test are presented in opposite directions, the amount of facilitation found should also be reduced. For a control group, presentation of ISIs up to 600 ms has no influence on coherence thresholds obtained (Raymond and Isaak, 1998).

Although difficulties shifting attention between the different objects (prime and test) presented in Experiment 2, could contribute to the results obtained, it is difficult to use this attentional process to explain the findings of Experiment 1 in which regardless of the stimulus parameters used, the group with dyslexia had higher coherence thresholds. However, when dot density was increased and longer stimulus duration used, normal temporal recruitment was found. These longer presentation times and high dot density might have allowed the group with dyslexia to orientate attention to the signal dots in the RDK more efficiently than in the conditions in which a low dot density and shorter stimulus duration was used. This would have allowed normal temporal recruitment. Findings of a larger effect size in between groups analysis when a low dot density is used in a global motion task provides some support for this conclusion (Benassi et al., 2010). In addition, presentation of stimuli of a higher contrast or different color to the noise dots might also have captured attention, allowing normal processing of coherent motion (Sperling et al., 2006; Conlon et al., 2012). The use of a pre-cue to direct attention either to low contrast motion signals in the RDK or to direct attention to specific features in a visual search task (Moores et al., 2011) has also provided evidence that directing attention can normalize coherence thresholds and improve visual search in groups with dyslexia. Although speculative, these results do indicate that attentional processes at PPC might influence coherent motion sensitivity, particularly when the computational complexity of the task is high.

The presence of a sensory processing deficit in the M or dorsal streams in groups with dyslexia is controversial (Ramus et al., 2003), with many studies presenting evidence that supports the presence of such a deficit (e.g., Cornelissen et al., 1995; Talcott et al., 2000). There are also studies that have found no evidence that a deficit is present (Ramus et al., 2003; Edwards et al., 2004; White et al., 2006) or have reported that about 30% of the group with dyslexia have this type of deficit (Amitay et al., 2002; Conlon et al., 2009). Alternative explanations such as difficulties with noise exclusion (Sperling et al., 2006), inattention (Williams et al., 2003) or temporal integration (Raymond and Sorensen, 1998) have also been presented. The results of the current study could be explained within the controversial sensory processing framework of dyslexia. The findings are supported by physiological evidence that neurons in the M and dorsal stream of groups with dyslexia are fewer in number, presented more sparsely and in a more disorganized manner than those found in normal readers (Galaburda and Livingstone, 1993; Stein and Walsh, 1997; Talcott et al., 2000) and that reduced neural activation at MT is found when VEP activity is measured during exposure to coherent motion (Schulte-Körne et al., 2004; Jednoróg et al., 2011). These findings are consistent with problems sampling the available motion signals present in the RDK, particularly when the perceptual difficulty of the task is high. However, these results might also indicate that directing and shifting attention between the feature specific components of these complex stimuli also contributes. Further research should directly investigate the extent that groups with dyslexia can optimize the perceptual filters needed to process complex stimuli and exclude noise, processes that can be evaluated within the perceptual template model of attention (Lu and Dosher, 2008).

Reading is also a computationally complex process which requires the use of a combination of visual, auditory and linguistic processes. Some researchers have suggested that the attentional consequences of impaired processing in the M and dorsal streams, causes difficulties with attention processing at PPC (Hari and Renvall, 2001; Vidyasagar and Pammer, 2010). Attentional difficulties have been linked to the way that children and adults with dyslexia process the sequentially presented letters on pages of text (Vidyasagar, 1999). Due to the sample sizes used in the current experiments, particularly Experiment 2, the associations between the sub-skills of reading and coherence thresholds were not evaluated. The systematic increases and decreases in coherence thresholds found in the group with dyslexia when different methodologies are used indicates that the processes need to efficiently perform a coherent motion task in adults with dyslexia can be normalized under specific circumstances. The challenge for future research is to determine whether the motion processing deficit found in dyslexia occurs in some individuals because of a vulnerability that is independent of their reading difficulties, or whether the deficit found is associated with the development or maintenance of word reading difficulties in this group. To make causal associations prospective longitudinal studies are needed, which measure temporal processing prior to the development of reading skills.

## Conflict of Interest Statement

The authors declare that the research was conducted in the absence of any commercial or financial relationships that could be construed as a potential conflict of interest.
